# Current innovations in head and neck cancer: From diagnostics to therapeutics

**DOI:** 10.32604/or.2025.060601

**Published:** 2025-04-18

**Authors:** TAYYABA SATTAR, IQRA NAZIR, MEHREEN JABBAR, JAVARIA MALIK, SABA AFZAL, SANA HANIF, SEYED ALI MOSADDAD, AHMED HUSSAIN, HAMID TEBYANIYAN

**Affiliations:** 1 University Institute of Physical Therapy, The University of Lahore, Lahore, 54000, Pakistan; 2 Faculty of Pharmacy, The University of Lahore, Lahore, 54770, Pakistan; 3 Department of Research Analytics, Saveetha Dental College and Hospitals, Saveetha Institute of Medical and Technical Sciences, Saveetha University, Chennai, 600077, India; 4 School of Dentistry, Edmonton Clinic Health Academy, University of Alberta, Alberta, T6G 1C9, Canada

**Keywords:** Head and neck cancer (HNC), Diagnostics, Therapeutics, Innovations

## Abstract

**Background:**

Head and neck cancers (HNC) account for a significant global health burden, with increasing incidence rates and complex treatment requirements. Traditional diagnostic and therapeutic approaches, while effective, often result in substantial morbidity and limitations in personalized care. This review provides a comprehensive overview of the latest innovations in diagnostics and therapeutic strategies for HNC from 2015 to 2024.

**Methods:**

A review of literature focused on pe-reviewed journals, clinical trial databases, and oncology conference proceedings. Key areas include molecular diagnostics, imaging technologies, minimally invasive surgeries, and innovative therapeutic strategies.

**Results:**

Technologies like liquid biopsy next-generation sequencing (NGS) have greatly improved diagnostic accuracy and personalization in HNC care. These advancements have improved survival rates and enhanced patients’ quality of life. Personalized therapeutic approaches, including immune checkpoint inhibitors, precision radiation therapy, and surgery, have led to enhanced treatment efficacy while reducing side effects. The integration of AI and machine learning into diagnostics and treatment planning shows promise in optimizing clinical decision-making and predicting treatment outcomes.

**Conclusion:**

The current innovations in diagnostics and therapeutics are reshaping the management of head and neck cancer, offering more tailored and effective approaches to care. Overall, the continuous integration of these innovations in clinical practice is reshaping HNC treatment and improving patient outcomes and survival rates. Future research should focus on further refining these technologies, addressing challenges related to accessibility, and exploring their long-term clinical benefits in diverse patient populations.

## Introduction

It is estimated that 630,000 cases of head and neck squamous cell carcinoma (HNSCC) occur worldwide each year, resulting in more than 350,000 deaths. In addition to differing in etiology, prognosis, and genomic alterations, HNSCC can also be classified based on the location of the tumor’s origin. Most HNSCCs occur in the mouth and oropharynx. Over 300,000 people are estimated to die of oral squamous cell carcinoma (OSCC) worldwide each year, with 145,400 estimated to die of it. After initial treatment, recurrence is expected for 32%–50% of patients, ranging from 50% to 60% [1–4]. Smoking and alcohol consumption are associated with squamous cell carcinomas of the oral cavity, pharynx, and larynx. In contrast, squamous cell carcinomas of the oropharynx are mostly associated with human papillomavirus infection, especially in young people and non-smokers. Men are more likely to suffer from this disease than women. In 2017, the World Health Organization (WHO) described tobacco-related and HPV-induced cancers as having different oncogenic pathways and prognosis. Sinus cavities and nasal fossae are rare anatomical locations for head and neck cancers, which are more likely to result from professional or environmental factors [5,6].

HNCs are typically accompanied by symptoms from the primary site, such as persistent hoarseness, long-term dysphagia, ulcers of the oral mucosa, epistaxis, or otalgia. First presenting symptoms of tongue base, upper glottis, or nasopharyngeal carcinoma include cervical lymphadenopathy. Sixty percent of patients (60%) are diagnosed at an advanced stage of the disease (III or IV) when survival rates are lower than at earlier stages (I or II). It is estimated that only 10% of patients with upper aerodigestive tract tumors will present with distant metastases at presentation, while 10%–15% will present with concomitant or delayed second primary tumors. 50%–60% of patients with locally advanced disease develop loco-regional recurrences, and 20% progress to distant metastases despite recent advances *in loco*-regional treatment protocols, such as advanced surgery, radiotherapy, and chemotherapy. As part of HNC care, there are many challenges, including the detection of primary tumors early on in the disease, the development of improved surveillance methods after potentially curative treatment, the identification of metastasis from recurrences and secondary primary tumors, and the development of therapeutic options for untreatable cases. Molecular advances in cancer biology have led to the development of treatments targeted against oncogenic genes and signaling pathways as major steps in the treatment of cancer [7–9]. Symptoms appear, but it takes a definite amount of time for a diagnosis to be made (histologically and radiologically). Generally, diagnosis delays are categorized into two phases: the patient delay, which is the time between the onset of symptoms and the point at which a patient seeks medical attention, and the system delay, which is the time elapsed between the initial contact with a healthcare professional and the definitive diagnosis/treatment [10,11]. A surgical resection is currently the only treatment option for HNSCCs or OSCCs. The outcome of resection can be good for cosmetics as well as survival if it is detected early. There has been some speculation that OSCC progresses from atypia to dysplasia to invasive malignancy, but most cases are found when they are already advanced. Extensive reconstructions may be required following surgery, as well as specialized postoperative care [1,2,12]. In this review article, a comprehensive overview of the current innovation in the field of head and neck cancer, from diagnostics to therapeutics, was reviewed.

## Methods

**The objectives of the study:** A comprehensive literature search was conducted to identify relevant studies, reviews, and reports on the latest innovations in head and neck cancer diagnostics and therapeutics.

**Literature sources and data gathering:** The databases searched included PubMed, Scopus, Web of Science, and Google Scholar. Articles published between January 2015 and October 2024 were considered to ensure a focus on current advancements. Keywords such as “head and neck cancer,” “innovations,” “diagnostics,” “therapeutics,” “immunotherapy,” “targeted therapy,” “biomarkers,” “robotic surgery,” “radiotherapy,” and “nanotechnology” were used in various combinations. Only peer-reviewed articles and conference proceedings in English were included in the review.

**Inclusion and exclusion criteria:** Articles were included if they met the following criteria: published between January 2015 and October 2024, focus on diagnostic and therapeutic advancements specific to head and neck cancer, reported on the clinical application of new technologies or therapeutic approaches, included studies that discuss novel biomarkers, imaging techniques, surgical technologies, immunotherapies, and drug delivery systems. Exclusion criteria included articles focusing on non-head and neck cancer cases, studies not involving human subjects or without clinical relevance, and publications that did not offer new or innovative information on diagnostic or therapeutic interventions. Potential limitations of this review include publication bias, as the review only considered published peer-reviewed studies and is limited to English-language publications, potentially excluding valuable research from non-English-speaking regions. The initial search resulted in 7285 articles, and then 2730 records were excluded upon removing duplicates, and 4340 records were excluded by title and abstract. A total of 140 records were excluded after analyzing the full text of 215 remaining articles. Seventy-five studies were included to structure this review article.

**Quality assessment criteria to establish why the paper was considered for inclusion:** The article directly addresses head and neck cancer (HNC), which aligns with your area of interest. It highlights current innovations in diagnostics and therapeutics for HNC, covering molecular diagnostics, imaging, minimally invasive surgery, and novel therapies. Discusses cutting-edge advancements that add a forward-looking perspective to the field. It covers diagnosis, staging, and treatment improvements, offering a holistic view of advancements in HNC care. Emphasizes patient-centered outcomes, such as enhanced treatment efficacy, reduced side effects, and improved quality of life. Addresses the growing incidence and burden of HNC, demonstrating relevance to public health. Uses data from trusted sources, including peer-reviewed journals and clinical trial databases. Focuses on recent developments, ensuring that the information is current and applicable.

## Results

### Diagnosis

#### Diagnosis imaging and biopsy

A thorough history is taken, and a physical examination is conducted before large biopsy specimens are obtained to avoid possible anatomical distortion caused by biopsy or false positive positron emission tomography results caused by biopsy. Initial histological diagnoses can be accurately made with fine-needle aspiration biopsy. The complete nodal resection of cervical nodes is recommended to prevent extracapsular metastatic spread and tumor spillage, which would require more radical treatment. Genetic biomarkers play a crucial role in head and neck cancer (HNC) diagnostics through biopsies, aiding in early detection and personalized therapy. Recently discovered biomarkers include TP53 mutations, PIK3CA amplifications, CDKN2A deletions, and NOTCH1 mutations. Emerging biomarkers like HPV DNA and circulating tumor DNA (ctDNA) offer non-invasive diagnostic potential, enhancing precision oncology approaches [13,14]. As part of the diagnostic process, it is crucial to provide a thorough clinical history that includes toxic and sexual habits, as well as a thorough physical examination that includes a thorough examination of the head and neck. An imaging diagnosis is necessary to explore tumor extension: An imaging diagnosis is required to avoid false diagnoses resulting from anatomy distortion resulting from a large biopsy. An examination of the cervical region by computed tomography or magnetic resonance imaging [15–17]. Magnetic resonance imagings (MRIs) provide superior imaging of the tongue, perineural spread, skull base invasion, and other anatomy. and extension of the brain within the skull cavity than CT imaging. Lymphatic dissemination is prognosticated by extracapsular nodal extension. X-rays or CT scans of the chest are preferred in the early stages of the disease. Using positron emission tomography- computed tomography (PET-CT), you can diagnose nodes (N), metastases (M), and primary tumors in synchrony. When definitive treatment is indicated or when CT or MRI scans suggest equivocal findings, it is recommended. In the case of dysphagia, an esophageal-gastric contrast study or an esophagoscopy may be recommended. Regardless of whether the tumor can be biopsied or not, fine needle aspiration (FNA) of lymph nodes is still required for histological diagnosis. In order to prevent the spread of extracapsular metastatic disease, complete nodal resection is preferred if a node biopsy is needed. The following functional abilities should be assessed: chewing, swallowing, phonation, breathing (airway stability should be assessed), odontology, and nutritional status. Psychological, social, and alcohol dependence evaluations, if necessary. For each patient’s treatment to be tailored, accurate staging is essential. It is important to note that a new classification system for p16-positive oropharyngeal tumors has been introduced: T4a and T4b have been combined into T4, and the N category has been redefined. Therefore, a downstaging occurs. Lip and oral cavity T categories (T1-T3) indicate the depth of invasion. N3a and N3b are subcategories of non-HPV-related tumors based on the extranodal extension (the absence of extranodal extension is specified in N1 and N2). A squamous cell carcinoma of the skin that invades perineural or deep becomes malignant [18–20]. Recent pan-cancer biomarker study has provided significant insights into identifying molecular signatures with potential applications for head and neck cancers (HNCs). These studies leverage genomic, proteomic, and immunological data to pinpoint diagnostic, prognostic, and predictive biomarkers, which could enable early detection and personalized treatment strategies [21]. MiRNAs such as miR-21-5p, miR-31-5p, and miR-155-5p have been implicated in tumor progression and survival outcomes across various cancers, including HNCs [22]. Despite the promise, studies face several challenges (limitations of current pan-cancer studies): The molecular diversity of HNCs, driven by factors such as HPV status and environmental influences, complicates the generalizability of biomarkers identified in pan-cancer studies. Many studies use limited or specific cohorts, which may not fully represent the global population of HNC patients. Biomarkers identified through bioinformatics often lack functional validation, limiting their immediate clinical application. High costs and the complexity of advanced diagnostic technologies restrict their widespread adoption, particularly in resource-limited settings. These limitations underscore the need for large-scale, multi-cohort studies and translational research to validate and optimize biomarker utility in HNCs. Such efforts could bridge the gap between biomarker discovery and clinical implementation, advancing personalized medicine for these complex cancers [23–25].

#### Exhaled breath analysis

Developing non-invasive approaches to cancer early detection is a crucial research topic. Samples can be taken by brushing the surface, by collecting saliva, or by drawing blood. Essentially, these approaches aim to identify cancer biomarkers or signatures that can be used to detect cancer before clinical symptoms appear. This early diagnosis could improve clinical outcomes by identifying the disease, planning treatment, and intervening at an early stage. Most cancers are diagnosed routinely by evaluating macroscopic appearance, radiological features, and histopathological characteristics. Due to the invasiveness, expense, and time involved in such diagnostic examinations, they cannot easily be used in population screening. Many cancers may be detected non-invasively, rapidly, and inexpensively by analyzing exhaled breath. Breath analysis has the advantage of having easy access to sample supply. Exhaled breath analysis is a non-invasive diagnostic method for detecting head and neck cancer (HNC). It focuses on identifying volatile organic compounds (VOCs) in breath, which are metabolic byproducts of tumor cells. Advanced techniques like gas chromatography-mass spectrometry (GC-MS) and electronic nose (e-nose) devices analyze VOC profiles, differentiating cancerous from healthy samples. This approach offers early detection, real-time monitoring, and high patient compliance due to its simplicity. Breath analysis also holds potential for personalized medicine by identifying cancer subtypes and tracking treatment response. Its accuracy, combined with non-invasiveness, makes it a promising tool for HNC diagnosis and management [26,27]. Proposed biomarkers can be seamlessly integrated into clinical workflows through non-invasive and cost-effective diagnostic techniques, such as saliva sampling, blood tests, and breath analysis. These methods offer rapid, accessible, and patient-friendly options compared to traditional invasive procedures like histopathological examinations. For example, breath analysis, which requires minimal training and infrastructure, could be implemented in routine clinical visits or community health screening programs. Biomarkers identified in these samples could guide early detection, allowing clinicians to diagnose cancers before symptoms appear. This integration would enable timely treatment planning, improving patient outcomes while reducing the reliance on expensive and time-consuming diagnostic methods [26,27].

#### Exosomes

Exosomes, nano-sized extracellular vesicles, play a critical role in head and neck cancer (HNC) detection and diagnosis due to their ability to carry tumor-specific biomarkers such as proteins, DNA, RNA, and miRNAs. Derived from cancer cells, exosomes reflect the molecular and genetic profile of tumors. Liquid biopsies can capture these exosomes from bodily fluids like saliva, plasma, and urine, providing a minimally invasive diagnostic approach. Their high stability and specificity make them ideal for early detection, monitoring disease progression, and assessing treatment responses. Exosome-based technologies, such as miRNA profiling, offer promising avenues for precision medicine in HNC management. Immune suppression in HNSCC is mediated in part by exosomes. Extracellular vesicles (EVs) are released by all types of cells and aid in communication between them. Due to their biogenesis process in the endosomal compartment, exosomes are distinguishable from other EVs due to their unique origin-specific cargo. Patients with HNSCC have plasma enriched in exosomes because tumors, including HNSCC, produce exosomes avidly. Located within the tumor microenvironment, exosomes play a crucial role in suppressing immune responses and regulating tumor growth. The unique biogenesis of exosomes, their ability to circulate freely in body fluids, and their molecular cargo have made them promising non-invasive liquid biomarkers. In HNSCC, exosomes can be used not only to monitor disease activity and tumor stage but also to monitor immune suppression, treatment response, and treatment outcome [28,29].

#### EVs as a potential biomarker of HNCs

**Figure 1:**
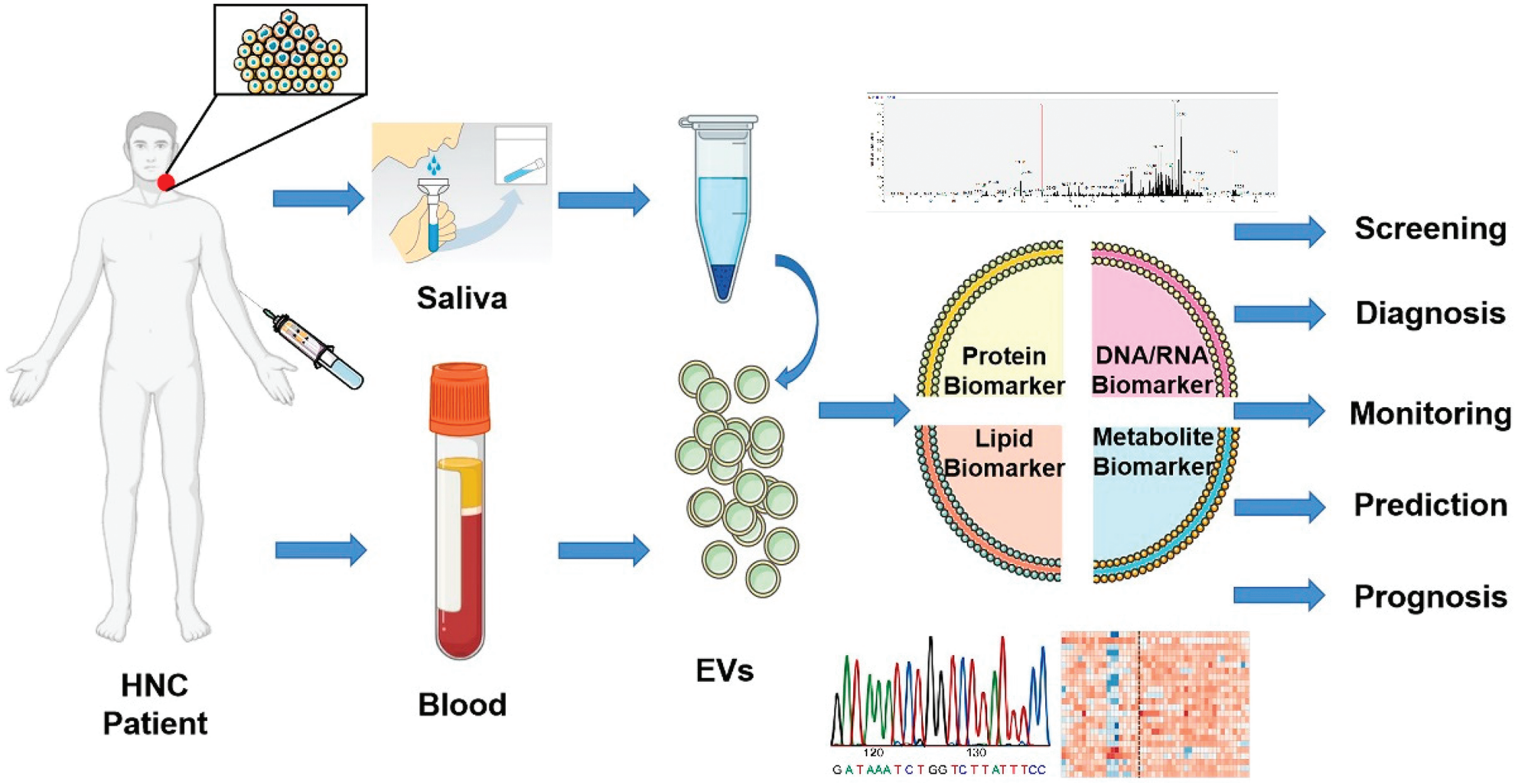
Extracellular vesicles have potential as biomarkers. The use of liquid biopsies derived from EVs can offer patients with head and neck cancer a non-invasive screening, diagnosis, monitoring, prediction, and prognosis method. With multi-omics studies, saliva or blood EVs can be analyzed, offering tremendous potential for improving patient care [30].

Extracellular vesicles (EVs) are small membrane-bound particles released by cells into body fluids, including blood, saliva, and urine. In the context of head and neck cancers (HNCs), EVs have gained attention as potential biomarkers for detection and diagnosis. These vesicles contain a variety of molecular cargo, including proteins, lipids, and nucleic acids (such as microRNAs and mRNAs), which reflect the physiological state of the originating cells. EVs derived from HNC cells carry specific molecular signatures that can help identify tumor presence and characteristics. Analysis of EVs from patient samples enables non-invasive monitoring, offering advantages over traditional biopsy methods. They also provide insight into tumor progression, metastasis, and treatment response. The high sensitivity and specificity of EVs as biomarkers hold great promise for early detection and personalized treatment strategies, potentially improving the prognosis and survival rates for patients with HNC. It is not possible to monitor the burden of HNC, the therapeutic response, or the potential for metastatic spread using biomarkers. As a result, a more sensitive, specific, economical, and robust method of detection is urgently needed (Fig. 1). Every cell, including tumor cells, secretes EVs that resemble, to a certain extent, their parent’s molecular cargo. In spite of the fact that biofluids are a non-invasive source of EVs, it is important to acknowledge that plasma, saliva, and other body fluids contain EVs derived from the host. There are also circulating tumor cells (CTCs) and circulating free DNA (cfDNA) as alternatives to EVs. However, EVs are pro-actively released by tumors and other cells rather than passively released through apoptosis, as lipid bilayers protect them. EVs are small and highly permeable to the extracellular matrix, and EVs contain a variety of molecular cargoes that are ideal for finding tumor markers [30,31]. Transcriptomes are composed of all transcripts in a cell that encode proteins, including coding (mRNA) and non-coding (small nucleolar RNAs, small nuclear RNAs, long-noncoding RNAs, and microRNAs). In addition to revealing the functions of genes, it also provides information about cell and tissue molecular structure, development, and disease. In clinics, RNA quantification levels are not consistently used as biomarkers due to a lack of consistent research. A lack of multicenter studies and cohorts with sufficient power could cause a lack of reproducibility. A wide variety of techniques, such as different sample types and sources, is also used [15].

#### Salivary protein biomarkers

Salivary protein biomarkers have emerged as a non-invasive and cost-effective tool for the detection and diagnosis of head and neck cancers (HNCs). Saliva, rich in proteins secreted by glands, contains biomarkers such as cytokines, enzymes, and growth factors linked to cancer development. Specific proteins like interleukin-8 (IL-8), vascular endothelial growth factor (VEGF), and matrix metalloproteinases (MMPs) have been identified as potential indicators of HNCs. These biomarkers are easily accessible through saliva collection, providing a painless alternative to invasive diagnostic techniques like biopsies. Advanced technologies, such as mass spectrometry and ELISA, enable precise quantification of these proteins, enhancing early detection and treatment outcomes. The majority of saliva consists of water; less than 1% contains electrolytes, proteins, and low molecular components. A salivary gland is one of the three major salivary glands in the mouth, and there are two minor salivary glands. There are many components of saliva, such as gingival crevicular fluid, viruses, bacteria, epithelium, erythrocytes, leukocytes, and food debris, that can be found in saliva. Saliva is also known as ‘whole saliva’ or ‘total saliva.’ The presence of proteins in saliva can be used as a biomarker to monitor treatment response or to detect early-stage tumors. The non-invasive properties of saliva make it an attractive biofluid for diagnosing HNSCC, and its biomarkers have been extensively studied. Furthermore, saliva is in the head and neck region, which means the tumor’s biomarkers would be released primarily through saliva. In biomarkers, a disease or condition is detected or confirmed in order to identify patients who have a subtype of it. Biological or pathological markers can be defined as alterations in cellular, biochemical, molecular, or genetic characteristics that can be detected and measured easily, whether in tissues, cells, or fluids. Detecting early disease recurrences requires that the selected biomarker shed light on the progression of the disease in a patient post-treatment. Further, the ideal biomarker should be highly sensitive and specific, preferably with minimal overlap between a cohort of patients and a control group. The specificity of a biomarker will depend on the clinical application, such as in a screening trial, where sensitivity is more important than specificity [32]. A salivary gland secretion contains saliva, gingival crevicular fluid, plaque, bacteria, nasal and bronchial secretions, lining cells, and blood. It also contains exogenous substances. In addition to protecting the oral mucosa from biological, mechanical, and chemical factors, saliva also plays an important role in the perception of oral sensations, lubrication, chewing, swallowing, and digestion. It also plays an important role in protecting the oral mucosa from bacteria, viruses, and fungal infections. Physiological data can be collected quickly, inexpensively, and non-invasively and can provide insight into an individual’s current physiological state. In addition to signaling oral disorders, salivary biomarkers also signal distal pathologies, suggesting that oral fluids may contain a substantial amount of molecular and microbial information that can be used to detect diseases throughout the body. Oral tumors can shed cellular material directly into saliva, providing a ‘sampling matrix’ for many molecular and physiological processes and reflecting abnormal pathology in distant organs. In light of the potential clinical use of this body fluid, a growing number of studies are being conducted to elucidate and develop saliva-based biomarkers for local and systemic diseases. Additionally, salivary biomarkers offer the potential for real-time disease monitoring and personalized treatment plans. By correlating the levels of specific biomarkers with tumor progression or treatment response, clinicians can make informed decisions regarding therapeutic interventions. Compared to blood or tissue biopsies, saliva-based diagnostics are advantageous due to their simplicity, minimal processing requirements, and lower costs. Integrating these biomarkers into clinical practice could significantly improve the early detection rates and overall prognosis of HNCs, ultimately benefiting patient care and reducing the burden of the disease [15].

#### Plasma and serum as sources for liquid biopsy

Plasma and serum are widely used as sources for liquid biopsy in head and neck cancer (HNC) detection and diagnosis due to their accessibility and rich biomarker content. Plasma is the cell-free component of blood containing circulating tumor DNA (ctDNA), cell-free RNA (cfRNA), proteins, and extracellular vesicles (EVs) like exosomes, which harbor tumor-specific genetic and epigenetic information. These biomarkers enable non-invasive detection of genetic mutations, epigenetic alterations, and aberrant protein expression associated with HNC. A nutrient delivery medium, plasma, serves as a vehicle to transport waste products from various organs of the body for excretion and transports nutrients to the cells. As well as transporting blood cells, it contributes to the maintenance of normal blood pressure. Upon centrifugation, all cellular components of whole blood are removed, resulting in plasma, which is composed primarily of water (92%). In plasma, most of the dissolved solids are proteins, while the remaining 1% contains glucose, lipids, enzymes, vitamins, hormones, and waste products such as urea and carbon dioxide. It is possible to separate serum from clotted blood by using a clear liquid. Except for fibrinogens, which play a role in blood coagulation, it shares the same composition as plasma. All proteins not required for blood clotting, as well as any exogenous substances and antibodies, are found in serum. During centrifugation, cellular components are removed from the blood. Fibrinogen and clotting factors are present in plasma obtained from anticoagulated blood. A coagulated blood sample (clotted blood) does not contain fibrinogen. Serum, derived by removing blood cells and clotting factors, shares similar biomarker profiles with plasma but with some differences in composition, such as clotting factor-induced degradation of certain nucleic acids. Both are pivotal for early cancer detection, monitoring tumor dynamics, and assessing treatment response. Advances in high-sensitivity technologies like next-generation sequencing and digital PCR enhance their utility in identifying low-abundance biomarkers, making plasma and serum invaluable tools in precision oncology for HNC [15,33,34].

### Surgical treatments for HNSCC

Surgical treatment of head and neck cancers remains an integral part of multidisciplinary treatment, requiring not only technical skills but an increased understanding of the biology of the disease being treated. Currently, head and neck cancer surgeons work closely with pathologists, radiologists, radiation and medical oncologists, and other medical specialists to offer patients a multimodality treatment that not only controls tumors but also prevents functional problems afterward. The majority of resectable tumors of the oral cavity and T4a laryngeal/hypopharyngeal malignancies should be treated primarily with surgery. Additionally, for the majority of early (T1-T2, N0) laryngeal and hypo/oropharyngeal carcinomas, primary surgery may be the best course of treatment [35]. Especially when the disease recurs and becomes treatment-resistant, head and neck cancers present unique therapeutic challenges because of their anatomic location and remarkably complex biology. It remains a mutilating disease despite current advancements in treatment modalities with a high mortality rate. Clinical and translational research is therefore urgently needed to advance treatment technologies and tools that combine high therapeutic efficiency with beneficial functional outcomes. Surgical research in head and neck cancer, therefore, aims to understand how surgery affects the disease and its microenvironment, unravels the biology of disease recurrences and treatment failures, validates existing and novel surgical treatments, and develops surgical strategies to improve function. A preclinical surgical model of human head and neck cancer helps to understand the biology of the disease and how surgery affects it, whereas clinical trials validate surgery as a treatment modality [35,36]. Curative surgery for HNSCC is limited to resectable tumors that are resected with clear margins, typically achieved through radical resection of the tumor with 1 cm tumor-free margins to obtain locoregional control and with preserved function. Nowadays, patients with head and neck cancer are rarely treated with the traditional open surgical technique known as radical neck dissection. The spinal accessory nerve, the internal jugular vein, the submandibular salivary gland, the tail of the parotid salivary gland, the sternocleidomastoid muscles, and all of the lymph nodes in the anterior and posterior triangles would all be removed in this procedure. The cervical lymph nodes in levels I through V were only removed during the modified radical neck dissection, and the rest remained untouched. These days, selective neck dissection (levels I–IV) or supraomohyoid neck dissection (levels I–III) are increasingly frequently used to remove cervical lymph nodes while preserving non-lymphatic tissues [37].

Depending on the particular architecture and features of the cancer, either minimally invasive resection or classical open resection may be carried out. Due to the aesthetic limitations caused by the visible scars that this operation may produce, classical open surgery is less desirable for younger people. Transoral endoscopic head and neck surgery is a minimally invasive procedure that can be done with laser resection or traditional instruments to operate on the oropharynx through a transoral channel [38]. The particular methods employed include transoral robotic surgery (TORS) and transoral laser microscopy (TLM). The postoperative morbidity is greatly decreased because these can be carried out without making external skin incisions, negating the necessity for a mandibulotomy or transmandibular technique to obtain access. The surgical procedure can take less time than the transcervical method. These methods offer a greatly enlarged image of the tumor, enabling the tumor to be confidently removed. TLM is performed via direct laryngoscopy using a carbon dioxide (CO_2_) laser for surgical resection. At the tissue-laser contact, water absorbs the laser beam after it passes through the endoscope, converting it into thermal energy that enables precise tissue cutting. This kind of resection is carried out using flexible microsurgical tools that improve access to the resected area and a binocular microscope that permits an intimate view of the tumor [39]. TORS is performed via an endoscope, and two other tools that are inserted into the patient’s mouth are within the surgeon’s control. All of the robot’s tool movements are under the surgeon’s control. The robot’s arms are equipped with a variety of interchangeable tools, including an electrocautery tool and gripping forceps. An endoscope or camera is inserted via the patient’s mouth while the mouth remains open with the use of an appropriate oral retractor. Suturing structures in limited visibility locations is made possible by the robotic arms’ flexibility, which is not achievable with conventional approaches [40]. The former approach can cause cosmetic deformities and functional impairments, making it less desirable for younger, healthy, and socially active patients with HPV-positive HNSCC. Currently, TORS is considered a function-preserving alternative to chemoradiation, with or without neck dissection. In the hands of experienced surgeons, TORS has proven to be both effective and oncologically safe for certain cases of HNSCC [41].

### Non-surgical treatments for HNSCC

#### Pharmacological treatment

Epidermal growth factor receptor (EGFR) inhibitors have been the only significant advancement in pharmacological treatment for head and neck cancers over the past few decades. One of the most promising molecular treatments for head and neck squamous cell carcinoma HNSCC is targeting the EGFR. HNSCC is among the many cancers where EGFR is overexpressed, playing a pivotal role in tumor growth and progression [42]. EGFR activation initiates crucial signal transduction pathways that contribute to the carcinogenesis of HNSCC. To target this pathway, two primary classes of drugs are crucial: tyrosine kinase inhibitors (TKIs), such as gefitinib and erlotinib, and monoclonal antibodies (mABs), including cetuximab and panitumumab. mABs work by binding to the extracellular domain of EGFR, preventing ligands from activating the receptor, promoting EGFR internalization, and, in rare cases, inducing antibody-dependent cell-mediated cytotoxicity (ADCC) [43]. On the other hand, quinazoline-derived TKIs inhibit the kinase function by preventing ATP from attaching to the intracellular tyrosine kinase domain of EGFR, thereby blocking downstream signaling. While these EGFR inhibitors are initially effective in many patients, most eventually develop resistance during treatment due to innate or acquired unresponsiveness to the drugs [44]. Immunotherapies, which harness the body’s immune system to fight tumors, have gained significant momentum in recent years. These treatments could boost the host immune system and encourage tumor cell lysis and subsequent tumor regression while minimizing harm to healthy cells [45]. This surge of interest began with the discovery that the Cytotoxic T-lymphocyte Antigen (CTLA-4) blocking antibody, ipilimumab, improved overall survival in patients with metastatic melanoma. Two consecutive impulses are necessary for T-cell activation. First, specialized antigen-presenting cells (APCs) attach to T-cell receptors (TCRs) when antigens are presented in conjunction with the major histocompatibility complex (MHC) I or II. A costimulatory signal is necessary for the second stage, which entails translating TCR stimulation into T-cell activation. This is accomplished when B7 molecules on the surface of the APC attach to CD28 receptors on the surface of the T-cell. The expression of CTLA-4, an inhibitory molecule, on the T-cell surface follows. Through interactions with the same ligands, CTLA-4 competitively inhibits B7’s binding to CD28, blocking the costimulatory signal and reducing T-cell activation and proliferation [46–49]. An IgG1 completely human monoclonal antibody called ipilimumab suppresses CTLA-4, which increases T-cell activation. Ipilimumab was created clinically following preliminary preclinical research that confirmed proof of concept showing antibodies against CTLA-4 might cause tumor regression. Advanced melanoma has seen the most clinical advancement [50]. T-cell inhibitory receptors, which serve as immunological checkpoints in charge of preserving the equilibrium between immune response activation and inhibition, are the target of the current immune checkpoint blockade of HNC. As a key resistance mechanism, tumors have mastered the use of immunological checkpoints. CTLA-4, PD-1, and other inhibitory receptors serve as buffers against the risk of excessive, potentially harmful T-cell activation. Both the quantity and the inhibitory activity of these T cell receptors are increasing in the tumor microenvironment (TME) [51]. Over the past decade, research efforts have expanded rapidly, focusing on understanding additional checkpoint pathways, identifying potential response targets, discovering novel mechanisms to induce tumor-specific immune responses, and uncovering biomarkers that predict treatment efficacy. Notably, recent clinical trials have shown that checkpoint inhibition with anti-PD-1 and PD-L1 antibodies could reactivate cytotoxic T cells to work against cancer cells, such as nivolumab and pembrolizumab, is effective in treating recurrent or metastatic HNSCC [1,52,53].

In addition to the PD-1/PD-L1 and CTLA-4 pathways, other inhibitory mechanisms have also shown promise in immunotherapy, including receptors with intracytoplasmic tyrosine (ITIM)-based inhibitory motives. These receptors can draw in phosphatases, such as Src homology region 2-containing protein tyrosine phosphatase (SHP)-1/2 or Src homology two domain-containing inositol 5′-phosphatase (SHIP), which transmit the inhibitory signal to effector immune cells (ICs) [54]. NK group 2 member A (NKG2A) is a subgroup of CD8+ T cells and an ITIM transporter receptor that is expressed in NK cells as a heterodimer with CD94. Alpha chain E (HLA-E), a non-classical HLA class I histocompatibility antigen that is overexpressed in these tumors, interacts with NKG2A/CD94 to produce its immunosuppressive impact by blocking the effector activities of CD8+ T and NK cells following phosphatase SHP-1 recruitment [54]. Therefore, mAb monalizumab increases the cytotoxic activity of NK and T CD8+ cells by blocking the binding of NKG2A/CD94 to HLA-E, which is typically stimulated by anti-tumor activity and Interferon-gamma (IFN-γ) [55]. According to Kuroki et al. (2023), beta-hydroxy-beta-methylbutyrate (HMB), arginine, and glutamine (Gln) were studied for their prophylactic effects. According to their findings, HMB/Arg/Gln administration inhibited the progression of grade 3 mucositis and cancer cachexia in HNSCC patients undergoing platinum-based concurrent chemoradiotherapy (PBCRT). An expanded phase III study is recommended [56].

#### Therapeutic vaccines

Most malignant cells overexpress the HPV oncogenes E6–7, providing an opportunity for the development of therapeutic vaccines. A combination of these vaccines with other immune checkpoint inhibitors (ICIs) is being investigated for their therapeutic applications in HNSCC for better clinical outcomes. Besides viral-based vaccines, other therapeutic vaccine strategies are also being researched, such as live vector-based vaccines, DNA/RNA-based vaccines, Dendritic cell (DC)-based vaccines, and peptide-based vaccines. A live vector-based vaccine can trigger an innate or adaptive response by using bacteria such as lactobacilli or viruses such as adenovirus. DNA/RNA strands and lipid carriers combine to form gene-based vaccines, which activate innate responses via immune receptors by acting as immunogenic agents. Due to their ease of preparation and stability, DNA-based vaccines are preferred over RNA-based vaccines [57].

#### Chemotherapy

Chemotherapy, the administration of anti-cancer or “cytotoxic” medications, works by targeting rapidly proliferating cancer cells and disrupting their life cycle, preventing their growth. Chemotherapeutic regimens often combine multiple agents, as this approach is more effective in inducing cell death compared to single-agent therapy. Different drugs work at various stages of the cancer cell’s life cycle, and when used together, they can enhance each other’s ability to trigger cell death. Similarly, the concurrent use of chemotherapy and radiation therapy in mixed-modality treatment can have a synergistic effect, boosting tumor cell death by amplifying each other’s actions on cellular replication machinery [58].

For patients with HNSCC in North America and Europe, chemotherapy—particularly platinum-based agents are commonly prescribed. Regimens approved by the National Comprehensive Cancer Network (NCCN) for treating oral squamous cell carcinoma (OSCC) and other HNSCC tumors include combinations such as carboplatin, paclitaxel, and 5-fluorouracil (5-FU). While many patients show an initial positive response to these treatments, most ultimately develop resistance over time. Additionally, cisplatin, a widely used chemotherapeutic agent for HNSCC, is a non-selective drug that can cause significant adverse side effects, limiting its long-term effectiveness [1]. Since the head and neck area is especially well-suited to regional chemotherapy, intra-arterial chemotherapy (IA chemotherapy) has been utilized for over 50 years to treat localized malignant neoplasms in patients with head and neck cancer. This approach allows for a more concentrated delivery of chemotherapeutic agents directly to the tumor site, potentially maximizing the therapeutic effect while minimizing systemic toxicity. However, creating and preserving artery access for these treatments presents significant challenges, which have been mitigated through major advancements in vascular radiology techniques. The development of novel devices, including fluoroscopy units and angiographic catheters, has made super-selective intra-arterial chemotherapy safer, more accurate, and repeatable [59]. In intra-arterial chemotherapy, achieving a higher infusion rate may result in greater drug concentration and more adequate dispersion of chemotherapeutic agents across malignancies. For the treatment of malignant brain tumors, skull base tumors, and head and neck tumors, we have employed three distinct injection techniques to increase the infusion rate: high-flow injection, high-dose injection with detoxification, and the flow-controlled injection method. These techniques help optimize the delivery and efficacy of the treatment. A promising therapy for advanced head and neck cancer involves intra-arterial infusion of high-dose cisplatin, with systemic neutralization by intravenous sodium thiosulfate (RADPLAT). This approach has shown potential benefits [59,60]. However, a Dutch trial comparing intra-arterial and intravenous chemoradiotherapy for advanced head and neck cancer found that RADPLAT was not superior to intravenous chemoradiotherapy [59,60]. Consequently, further research on RADPLAT is necessary to define its indications better and to improve treatment outcomes [59,60].

#### Immunomodulatory targets for head and neck

Immunomodulatory therapy played an important role in tumor cell growth long before Ehrlich proposed it [57]. Herbermann discovered NK cells as a defense mechanism against malignancies in the body [61]. Additionally, patients with HNSCC who have undergone organ transplantation are more likely to develop tumors [57]. As immunosuppressive malignancy, HNSCC is a cancer that affects the immune system. This process is mediated by the tumor cells’ influence on the TME, which manipulates the immune system and promotes the production of immunosuppressive mediators. The tumor cells themselves are responsible for further immune suppression as they grow and advance in stage. Metastasis, angiogenesis, and, ultimately, tumor progression are all caused by this. This means that as tumors progress through HNSCC stages, immunosuppression increases [57,62]. Based on Vos et al.’s (2021) report, no major pathological response (MPR) patient developed recurrent HSNCC after 24.0 months of follow-up. MPR patients can be identified with FDG-PET based on their total lesion glycolysis prior to surgery. Patients with MPR have a baseline mutational profile associated with AID/APOBEC (a family of proteins that deaminate cytidine in DNA and RNA), as well as a reduction in hypoxia RNA signature after treatment. Neoadjuvant COMBO ICB (immune checkpoint blockade (ICB) combination therapy) has shown encouraging efficacy in HNSCC based on their study data [63].

### Photothermal therapy (PTT)

Near-infrared (NIR) is used as part of PTT to treat localized, minimally invasive conditions. By exposing tumor tissues to concentrated light, photosensitizers are activated to produce reactive oxygen species, which initiate cascades that eventually destroy the cells. Because photosensitizers and irradiating light are themselves essentially inert, PTT is much less toxic than chemo- or radiotherapy, making it suitable for repeated treatments of HNSCC patients. A considerable problem in PTT is photo-toxicity caused by the unintentionally systemic distribution of PS, as well as efficacy. In Europe, Foscan® is approved for the treatment of HNSCC in advanced or recurrent stages; it is a form of photosensitizers temoporfin formulated with ethanol/propylene glycol. Due to its poor tumor-selectivity and severe cutaneous burns, it was not approved for treating HNSCC in the U.S. For PTT to be used clinically, precise tumor-targeting must be improved [64]. The Multinational Association of Supportive Care in Cancer recommended Low-Level Lasertherapy (LLLT)/ Photobiomodulation therapy (PBMT), but Legouté et al. (2019) did not have the power to assess it. The LLLT/PBMT treatment was well tolerated and safe, making it an excellent treatment for severe oral mucositis (OM) [65].

### Radiation therapy

Radiation treatment for HNSCC has been around for nearly a century and can be provided for palliative care, as an adjuvant technique, or in a curative situation [66]. HNSCC is a complicated clinical condition, and finding a treatment plan is still difficult. A multidisciplinary team should consider the appropriate management practices for each patient with HNSCC. The current standard of care for the majority of patients treated definitively or adjuvantly following surgery in cases of locally advanced disease is radiation therapy (RT) with or without concurrent cisplatin-based chemotherapy [67]. Surgery can be reduced, tissue function can be preserved, and better cosmetic outcomes can be achieved through this method. Radiation dosing and administration have improved dramatically over the years, including intensity-modulated radiotherapy and brachytherapy. However, there are still adverse effects associated with radiotherapy, including tissue and bone necrosis [68]. Relapses in radiotherapy are also common, requiring additional radiation, surgery, or chemotherapy [1]. To evaluate the treatment volume and outline of tumor and normal tissues, physical examination and multimodality imaging rely on 3-dimensional anatomic details. CT, MRI, and PET-CT are examples of such imaging. It is possible to combine multimodality imaging. MRI can be combined with CT for a better understanding of soft tissues, and PET-CT for metabolic information. This volume includes the primary cancer site and the lymph nodes, which are considered gross tumor volumes (GTVs). In addition to the gross tumor volume, microscopic cancer involvement is also included, which is the clinical tumor volume (CTV1). Based on the microscopic evaluation of the surgical specimens, it is enlarged 2.5 to 10 mm to the planning target volume (PTV1). The typical dose is usually 66 to 74 Gy (Gray) in 2 Gy fractions or 81.6 Gy in 1.2 Gy fractions. The tumor will, therefore, be radiated from various angles and planes. By following the outline of the target, each beam is designed to converge and deliver the planned dose while only affecting normal tissue fractionally. Radiation therapy that wraps tumors with high doses while delivering a minimum dose to the surrounding normal tissues is called conformal radiation therapy. To reduce radiation-induced toxicities, advanced radiotherapy techniques were used, including intensity-modulated radiation therapy (IMRT), which is a new form of conformal radiation therapy (3D CRT) that changes radiation intensity across beams, whereas 3D CRT delivers radiation to the target with a minimum dose to adjacent tissues [69]. HN tumors require high-dose radiation to be administered to a small area that contains or is very close to many critical structures, such as the spinal cord, brainstem, brain optic pathways, brachial plexus, salivary glands, swallowing-related structures, and larynx. 3-D conformal RT and intensity-modulated RT (IMRT) are used in today’s curative-intent radiotherapy. A major advantage of IMRT is that it allows radiation oncologists to reduce unintentional irradiation of healthy tissues when compared to 3D conformal RT. Radiation oncologists have been using curative-intent IMRT extensively in clinical practice to improve oncologic outcomes, reduce radiation-related toxicity, and expand indications [33,70]. The Bragg peak and lack of exit dose of proton beam therapy are crucial factors in treatment planning with proton beam therapy. Planned beam paths to the target must be selected in such a way that they are short and reliable for the planning team and radiation physician. Despite the fact that protons offer a superior dose of homogeneity over photons, proton penetration may cause worse issues with tissue inhomogeneity. There is a risk of irradiation of normal tissue as a result of artifacts, such as dental or surgical hardware, which can make the Bragg peak difficult to locate, thus resulting in suboptimal treatment delivery. Beams should not go through mouths (dental artifacts), hollow organs, or critical structures (spinal cord, salivary glands) since these structures are important. The size and weight of tumors, as well as the position of patients during each day, can complicate treatment plans. A thorough quality assurance program and reimaging during treatment are essential for addressing all of these technical uncertainties and ensuring the treatment’s integrity. In addition, skin toxicity should be minimized by implementing three-dimensional conformal passive scattering techniques [71]. Based on Kiyota et al. (2022) findings, weekly cisplatin chemotherapy is not inferior to 3-weekly cisplatin chemotherapy for high-risk locally advanced squamous cell carcinoma of the head and neck (LA-SCCHN) patients. Based on these findings, it may be possible for these patients to receive chemoradiotherapy with weekly cisplatin [72]. It is hypothesized that the addition of stereotactic body radiation therapy (SBRT) to anti-PD-1 is safe, enhances pathological response, and is more effective than checkpoint inhibitors alone in patients with locoregionally advanced HNSCC. The study showed radiotherapy delivered to the gross tumor volume and immunotherapy combined to result in high mPR, which is recommended for localized neoadjuvant therapy [73]. Radiation therapy (RT) induced sialadenitis in head and neck cancer patients can be relieved by Botox, according to Nieri et al. (2023). The researchers observed no complications or side-effects when Botox was administered to the salivary gland prior to external beam radiation. Compared with controls, the Botox group did not further reduce salivary flow following RT. Inhibition of CXCL 1 at V3, which was reduced in the Botox group, might be a candidate for further investigation in radiation-induced sialadenitis [74].

### Exosomes as therapeutic vesicles

There is a high rate of drug resistance in patients with metastatic or recurrent HNSCC. It may be possible to increase the effectiveness of chemotherapy treatments, prevent chemoresistance, and reduce cytotoxic side effects by targeting the delivery of chemotherapeutics. Because exosomes have low immunogenicity, good biodistribution, and bioavailability, they are becoming increasingly popular as drug-delivery vehicles for chemotherapeutics and therapeutic short-interfering RNAs (siRNA) [28].

### EVs and treatment of HNCs

**Figure 2:**
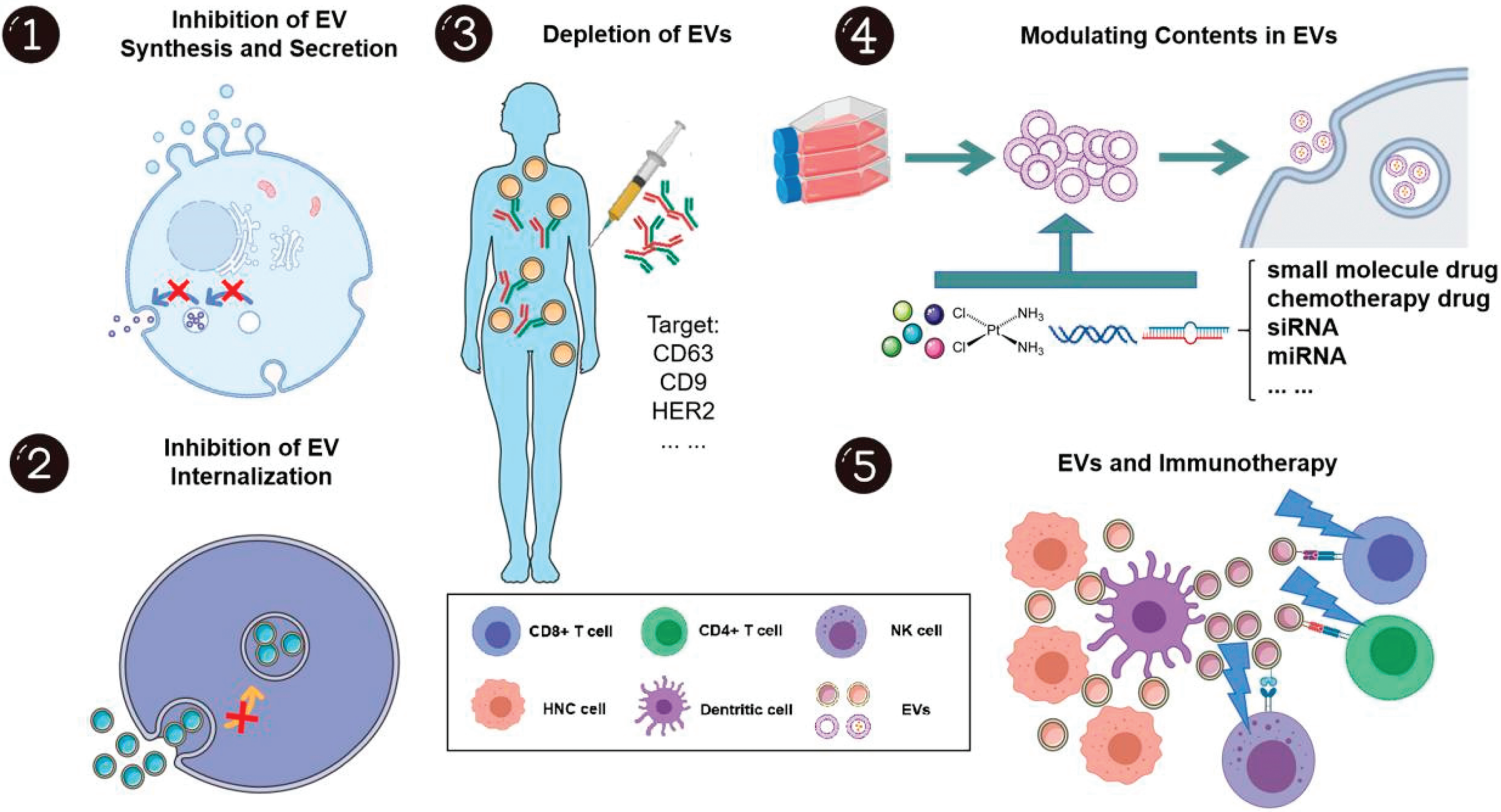
Using extracellular vesicles to treat head and neck cancer [30].

The role of EVs in the development and progression of HNC has led many researchers to consider them as therapeutic targets and novel drug delivery systems (Fig. 2). EVs are both excellent targets for treatment and excellent carriers for drugs. To some extent, blocking EV synthesis, secretion, internalization, and even depletion may be effective in preventing head and neck cancer development and progression. A major advantage of EVs is their bioavailability, biocompatibility, and ability to target drugs, making them ideal drug delivery systems. It is also possible that EVs disrupt immunosuppressive environments and can be used as immunotherapeutic “adjuvants” [30].

### Gene therapy and the p53 pathway

Patients with inherited diseases with monogenic mutations were initially studied with gene therapy in the 1990s and early 2000s. The two most common types are cystic fibrosis and hemophilia. Although researchers attempted gene therapy, their efforts mostly failed due to their inability to express genes permanently. Virus-mediated gene therapy has also been the subject of legitimate safety concerns, particularly after reports of leukemogenesis. p53, p16, p15, p27, and retinoblastoma gene (Rb1) are among the primary tumor suppressor genes altered in HNSCC. A significant percentage of patients with head and neck cancer are found to have mutations of the p53 gene. Having a p53 mutation increases the chances of disease recurrence, resisting chemoradiation, and having a high level of tumor invasiveness. Virus-mediated gene therapy faces limitations such as immunogenicity, where the immune system may attack viral vectors; limited cargo capacity for certain viral vectors; potential off-target effects; risk of insertional mutagenesis leading to cancer; challenges in targeting specific tissues; and high production costs, limiting its accessibility and widespread clinical application [75]. Gene therapy that restores the function of the p53 gene is commonly known as corrective gene therapy. The mechanism of gene therapy involves modulating the immune system to overcome immune tolerance created by tumor cells, possibly by increasing the presentation of tumor-associated antigens or increasing the expression of major histocompatibility complex I. It was originally thought that gene therapy could enhance immune function by adding cytokines to isolated tumor cells *ex vivo*. Due to the time and cost associated with autologous cell modification, these attempts became less popular. In recent years, the focus has shifted away from improving tumor immunogenicity and toward enhancing patients’ immune responses. The field of immunogene therapy is one of the most promising. T cell-mediated responses are being created by identifying and cloning tumor-associated antigens for enhanced immune surveillance [75].

## Discussion and Future Direction

The future of head and neck cancer management is poised for significant transformation as novel innovations continue to emerge across diagnostics, therapeutics, and personalized care. A few key areas expected to drive future advances include Liquid Biopsy and Early Detection: The use of circulating tumor DNA (ctDNA) and other liquid biopsy technologies will likely become more widespread, allowing for earlier detection of head and neck cancers, monitoring of treatment response, and identification of minimal residual disease. These non-invasive tools will help clinicians personalize treatment strategies and improve patient outcomes. Immunotherapy Advancements: While immunotherapy has already made strides in treating head and neck cancers, particularly with immune checkpoint inhibitors, ongoing research into new therapeutic targets and combination strategies is expected to yield more effective, durable responses. Future work will focus on optimizing patient selection for immunotherapy and overcoming resistance mechanisms. 3D Bioprinting and Regenerative Medicine: As surgical techniques advance, the future of post-surgical care for head and neck cancer patients may involve the use of 3D bioprinting and tissue engineering for reconstructive purposes. Regenerative medicine approaches could accelerate recovery, minimize scarring, and improve functional outcomes, enhancing the quality of life for survivors. Artificial Intelligence (AI) and Machine Learning: AI and ML hold enormous potential for enhancing diagnostic accuracy and treatment planning. Future developments may enable AI-powered imaging analysis to detect minute cancerous changes undetectable by the human eye. At the same time, ML algorithms could predict patient responses to specific treatments, facilitating personalized approaches and reducing unnecessary treatments. Nanotechnology in Drug Delivery: Nanotechnology-based drug delivery systems are anticipated to improve further the precision and effectiveness of chemotherapy and radiation therapies. Nanocarriers can deliver therapeutic agents directly to cancer cells, minimizing side effects and improving the therapeutic index. Gene Therapy and Targeted Therapies: The integration of gene therapy approaches has the potential to revolutionize head and neck cancer treatment. Targeting specific oncogenic mutations could offer more effective and less toxic treatment alternatives, improving outcomes for patients with advanced or resistant disease. Telemedicine and Remote Monitoring: Telemedicine and wearable technologies will play an increasing role in monitoring patients post-treatment. Remote follow-up care, integrated with AI-powered monitoring systems, will allow for earlier detection of recurrence and timely interventions, reducing the burden on healthcare systems and improving patient access to care. Precision Oncology and Multi-Omics Integration: As multi-omics technologies (genomics, proteomics, metabolomics) continue to evolve, integrating these data into clinical practice will lead to more personalized treatment approaches. Future research will likely focus on identifying specific biomarkers to predict treatment response and tailor therapies accordingly, ensuring that each patient receives the most effective treatment. These future advancements, driven by technology and innovation, will further optimize the management of head and neck cancers, improving early detection, treatment efficacy, and overall patient survival. With continued collaboration between clinicians, researchers, and technologists, head and neck cancer care will become more precise, personalized, and less invasive, offering hope for improved outcomes and quality of life for affected patients.

## Limitations

Many innovative diagnostic and therapeutic technologies are still relatively new, and long-term efficacy and safety data are often lacking. This limitation makes it difficult to fully assess their potential in improving survival and quality of life for patients with head and neck cancer. Advanced diagnostic tools and therapies, such as genomic profiling, precision medicine, and robotic surgery, may not be readily available in all healthcare settings, particularly in low- and middle-income countries. This disparity limits the global applicability and impact of these innovations. The cost of developing and implementing cutting-edge therapies, such as immunotherapy and targeted therapy, can be prohibitive. These expenses may limit patient access and create inequalities in treatment options, especially for those without comprehensive health insurance coverage. New diagnostic tools and surgical technologies, such as advanced imaging techniques and robotic surgery systems, require specialized training. Surgeons, oncologists, and other healthcare professionals may face a learning curve, which could temporarily affect the quality of care during the transition to these new methods. Some innovations, especially those involving genetic profiling or AI-driven diagnostics, may raise ethical concerns regarding patient consent, privacy, and data security. These concerns may limit the widespread adoption of such technologies in clinical practice. Head and neck cancers are heterogeneous, and responses to new therapies may vary significantly based on genetic mutations, tumor location, and patient-specific factors. This variability makes it challenging to develop a one-size-fits-all approach using the latest innovations. New therapies and diagnostic technologies often face regulatory challenges, which can delay their availability in the clinic. The lengthy approval processes may slow the adoption of these innovations and delay their benefits for patients. The integration of new diagnostic tools and treatments into established clinical workflows may take time, as it requires validation through clinical trials and adaptation to current standards of care. This process can slow the translation of innovative technologies from research to everyday clinical practice. The increasing use of advanced technologies in diagnostics and treatment may inadvertently lead to an over-reliance on machines and algorithms, potentially reducing the emphasis on clinical judgment and patient-centered care.

## Data Availability

All data generated or analyzed during this study are included in this published article.
